# An international multi-centre prospective study on the efficacy of an intraarticular polyacrylamide hydrogel in horses with osteoarthritis: a 24 months follow-up

**DOI:** 10.1186/s13028-015-0110-6

**Published:** 2015-04-15

**Authors:** Aziz Tnibar, Hans Schougaard, Linus Camitz, Jonas Rasmussen, Marc Koene, Werner Jahn, Bo Markussen

**Affiliations:** Department of Large Animal Sciences, Faculty of Health and Medical Sciences, University of Copenhagen, Hoejbakkegaard alle 5, DK-2630 Taastrup, Denmark; Noerlund Hestehospital, Roedebækvej 2, DK-8653 Them, Denmark; Camitz equestrian, Aasoe Mosevej 13, DK-4171 Glumsoe, Denmark; Hoejgaard Hestehospital, Rugaardsvej 696, DK-5462 Morud, Denmark; Tieraerztlische Klinik fuer Pferde, Essener Strasse 39a, D-49456 Bakum, Germany; Pferdeklinik Bargteheide, Alte Landstrasse 104, D-22941 Bargteheide, Germany; Department of Mathematical Sciences, Laboratory of Applied Statistics, University of Copenhagen, Universitetsparken 5, DK-2100 Copenhagen, Denmark

**Keywords:** Osteoarthritis, Horse, Treatment, Polyacrylamide hydrogel, 2 years follow-up

## Abstract

**Background:**

Polyacrylamide hydrogel (PAAG) was evaluated recently to treat osteoarthritis (OA) in horses with highly encouraging results; however no long term field-study was done to explore its clinical efficacy and lasting effect. The objective of this study was to evaluate the efficacy of PAAG in improving clinical signs of OA in horses. We hypothesized that lameness grade would significantly improve and the effect would last at least 2 years in osteoarthritic joints treated with PAAG. Forty three horses older than 2 years with OA in only one joint based on clinical evaluation, intra-articular anaesthesia and imaging (radiography) were included in this study. Horses were injected with 2 ml of PAAG into the affected joint and were followed up at 1, 3, 6, 12 and 24 months. Efficacy of PAAG was evaluated by blinded clinical assessment of lameness. Adverse reactions to joint injection were assessed. Data relating to case details, type of activity, joint and limb involved, lameness duration, lameness grading, previous joint treatment, joint effusion grading, radiographic grading, and owner assessment were recorded. Factors associated with the outcome measure “lameness grading” were analyzed using generalized linear mixed model for logistic regression.

**Results:**

At 1, 3, 6, 12 and 24 months follow-up, 59%, 69%, 79%, 81/% and 82.5% of horses were non-lame respectively. Reduction of joint effusion was observed over time. No side effect was observed in the treated joints. There was a significant decrease in lameness grade from baseline to 1, 3, 6, 12 and 24 months (*P* < 0.0001) and a significant positive association with joint effusion (*P* < 0.0001). Estimates for odds ratio (OR) showed that the effect of treatment increased over time (OR for lower lameness from month 1 to month 24 relative to baseline increased from 20 to 58).

**Conclusions:**

PAAG significantly alleviated lameness and joint effusion in osteoarthritic joints. PAAG is a safe and lasting (at least 24 months) OA treatment in horses. PAAG is a promising new treatment for OA in horses.

## Background

Osteoarthritis (OA) is a common clinical problem in horses [1,2] and is the most common joint disease and one of the most frequent causes of physical impairment in humans [3]. Surveys estimate that up to 60% of lameness problems in horses are related to OA [1], which can occur early in the equine athlete’s career or later in older horses [4]. The fetlock (metacarpo(tarso)phalangeal) joint is a common joint for spontaneous OA in horses [5].

As part of the OA-complex, elastoviscosity of the synovial fluid is abnormally low [6], and thus the use of visco-supplementation, for example intra-articular injections of high molecular-weight sodium hyaluronan (SH), has been implemented as part of the treatment for OA in humans [7,8], and horses [9].

Polyacrylamide hydrogel ^a^ (PAAG) is a non-toxic and non-immunogenic biocompatible polymer gel consisting of 97.5% sterile water and 2.5% cross-linked polyacrylamide [10,11]. Its biocompatibility in soft tissues (e.g. reconstructive surgery, urology) has been demonstrated [12-14]. Also, PAAG is a non-particulate homogenous gel similar to SH gel in overall structure and tissue compatibility [13], but with a longer-lasting viscous effect, as it is non-degradable [10]. This gel has been used for years in the augmentation of connective tissue in human medicine [14,15]. Experimental studies supported by histopathological observations have shown that PAAG exerts its effect via integration over time within the soft tissues, through a combination of vessel in-growth and molecular water exchange [10,13]. A recent clinical study investigated the effect of PAAG on improving clinical signs of equine OA within the metacarpo(tarso)phalangeal joint or one of the carpal joints (antebrachiocarpal, middle carpal or carpometacarpal) [16]. Thirty-three horses, older than two years with OA located within only one joint were treated intra-articularly with PAAG. At 1, 3, and 6 months after treatment, 81%, 88% and 87% respectively of horses showed improvement in lameness grade compared with baseline. At 6 months, approximately 70% of horses were non-lame [16]. At 12 months, 81% of the horses from the same study population were non-lame [17]. A recent pilot study using an experimental OA model in goats has shown that PAAG was integrated into the synovial membranes of the injected joint, and significantly improved the lameness caused by OA, with 75% (3 out of 4) of the cases becoming non-lame at 4 months post treatment evaluation [18]. A comparative prospective study has demonstrated that horses with OA treated with PAAG significantly improved their clinical signs when compared to horses with OA treated with triamcinolone acetonide combined with SH [19]. Another report has shown that PAAG effectively relieved lameness in horses with distal interphalangeal joint OA [20].

The purpose of this two year prospective clinical study was to investigate the efficacy and duration of action of PAAG for improving clinical signs of OA in the equine metacarpo(tarso)phalangeal or one of the carpal joints. Our hypothesis was that lameness scores would significantly improve and the effect will last at least 2 years in osteoarthritic joints after treatment with PAAG.

## Methods

The clinical study was conducted between October 2010 and February 2014 at 5 major equine hospitals (3 in Denmark, 2 in Germany). The study was approved by the National Council for Animal Experimentation (Authorization number: 2010/561-1890). All horse owners gave written informed consent. Client-owned horses older than 2 years with OA confirmed clinically within a single joint (metacarpo(tarso)phalangeal joint or one of the carpal joints (antebrachiocarpal, middle carpal or carpometacarpal)) were included in this clinical study. The confirmation of OA was based on clinical evaluation, lameness abolished after intra-articular anesthesia (10 ml of local anesthetic per joint, horses reexamined in 10 min) and imaging (radiography). Lame horses with severe radiographic abnormalities were also included in the study. Exclusion criteria in this study were horses with lameness problems localized in more than one joint, horses with OA secondary to joint infection, horses that had undergone surgery of this joint (including arthroscopy) within three months preceding the study, and horses with any other anti-arthritic treatment (e.g. nonsteroidal antiinflammatory drugs, corticosteroids, SH) administered to the affected joint within two months preceding the study. Other exclusion criteria included horses that had received any additional anti-arthritic treatment, or undergone surgery during the study period.

The study was designed as a prospective clinical study. This study incorporated horses described in previous reports [16,17]. At baseline (day 0), horses were injected with 2 ml of PAAG into the affected joint. In all cases, this injection was performed the same day as the intraarticular anesthesia. After treatment, horses were rested for the first two weeks with only 10 to 15 minutes hand walking exercise per day, then for the subsequent two weeks, all horses were allowed hand walking exercise for 20 to 30 minutes per day or turnout in a small paddock. All horses were clinically assessed under similar circumstances by clinicians (one per center) different from the one who had originally examined and treated the horse, and unaware of the identity of the horse and whether joints were treated or not at 1, 3, 6, 12 and 24 months post-treatment. All horses received only one injection of PAAG during the study.

Efficacy of the treatment was evaluated by lameness examination of the affected joint, including response to flexion tests. Each horse underwent lower limb (interphalangeal and metacarpo(tarso)phalangeal joints) and carpal flexion tests for 1 min for all limbs. Horses were evaluated in hand on a hard surface in straight lines and in circles. Data relating to case details, including type of activity, limb involved, lameness duration (1–6 months, >6 months), previous joint treatment (yes (type, duration); no), and lameness grade was collected at baseline. Lameness grading [21] was performed at baseline, and at 1, 3, 6, 12 and 24 months. Joint effusion grading (0: no distension, 1: mild, 2: moderate, 3: marked and 4: severe) was only visually assessed at baseline and at 1, 3, 6, 12 and 24 months. The radiographic grading of OA was based on standard radiographic projections [22] for each joint (0: no lesion, 1: mild, 2: moderate and 3: marked) at baseline only. The radiographic grading system used was described previously [23] and was used by a clinician experienced in radiography. The owner’s assessment of the result of the treatment (1: not satisfied, 2: slightly satisfied, 3: satisfied and 4: very satisfied) was recorded at 1, 3, 6, 12 and 24 months. Safety of the joint treatment was evaluated through recording of any adverse reaction following joint injection. If the horse was non-lame one month after post treatment, then the horse was allowed to progressively resume its normal activity.

### Statistical analysis

The statistical variables used in this study are described in Table 1. Variables potentially associated with the outcome measure “lameness grading” were analyzed using a generalized linear mixed model for ordinal regression with horse identification specified as a random effect. The initial model consisting of all main effects was reduced by backward model selection sequentially removing non-significant effects on a 5% significance level. To investigate the development of joint effusion a similar analysis was done using “effusion grading” as outcome. This analysis was done without including “lameness grading” as explanatory variable. The statistical analysis was done using SAS V9.4.

**Table 1:** **Variables included in the statistical analysis of the study population of 43 horses with osteoarthritis of a metacarpo(tarso)phalangeal joint or carpal joint treated by intra-articular administration of a polyacrylamide hydrogel**

**Variables**	
- Horse	43
- Sex	Female, male, gelding
- Breed	Warmblood, racing breed, others
- Type of activity	Dressage, jumping, racing, others
- Time point (months)	0, 1, 3, 6, 12, 24
- Joint	Metacarpo(tarso)phalangeal, carpus
- Limb involved	Front, Hind
- Lameness duration before treatment (months)	1 to 6, > 6
- Previous treatment	Yes, No, Unknown
- Radiographic grading of OA	0 to 3
- Joint effusion grading	0 to 4
- Lameness grading	0 to 5

OA: osteoarthritis.

## Results

A total of 43 horses met the inclusion criteria for this study. Only 41, 26 and 40 horses were examined at months 1, 3 and 24 respectively. Table 2 summarizes the descriptive data of the study population. At baseline, the proportion of horses with a moderate to marked radiographic grade was 47%, whereas 53% of horses had mild radiographic grade.

**Table 2:** **Description of the study population of 43 horses with osteoarthritis of a metacarpo(tarso)phalangeal joint or carpal joint treated by intra-articular administration of a polyacrylamide hydrogel and variables evaluated during a 24 months follow-up**

**Variable**	**Data**
Horses (no.)	43
Mean (range) age (years)	9.4 (2–15)
Breeds (no. (%))	
Warmbloods	30 (70%)
Racing breeds	8 (19%)
Others	5 (11%)
Horse activity (no. (%))	
Dressage	15 (35%)
Jumping	13 (30%)
Racing	8 (19%)
Other	7 (16%)
Limb involved (no. (%))	
Front	27 (63%)
Hind	16 (27%)
Joint involved (no. (%))	
Metacarpo(metatarso)phalangeal	40 (93%)
Antebrachiocarpal, Middle carpal, Carpometacarpal	3 (7%)
Lameness duration before treatment (no. (%))	
1–6 months	35 (81%)
>6 months	8 (19%)
Previous anti-osteoarthritic therapy	
Yes	37 (86%)
No	6 (14%)
Lameness grading at baseline (no. (%))	
1	11 (26%)
2	14 (32%)
3	15 (35%)
4	3 (7%)
Joint effusion grading at baseline (no. (%))	
0	3 (7%)
1	10 (23%)
2	18 (42%)
3	7 (16%)
4	5 (12%)
Joint effusion grading at 24 months (no. (%))	
0	31 (77.5%)
1	8 (20%)
2	0 (0%)
3	1 (2.5%)
4	0 (0%)
Radiographic grading of OA at baseline (no. (%))	
1 (mild)	23 (53%)
2 (moderate)	9 (21%)
3 (marked)	11 (26%)
Proportion of non-lame horses at (%)	
1 month	60%
3 months	67%
6 months	79%
12 months	81%
24 months	82.5%
Owner satisfaction at 24 months (%)	
Not satisfied	2.5%
Slightly satisfied	7.5%
Satisfied	15%
Very satisfied	75%

OA: osteoarthritis.

Lameness changes in relation to baseline lameness score. Before treatment (baseline), the proportion of horses with lameness grade 1, 2, 3 and 4 were 26%, 32%, 35% and 7% respectively. In horses with baseline lameness grade 1 (n = 11), 73% were non-lame at 1 month, and 60 to 82% were non-lame at the following controls (3, 6, 12 and 24 months). In these horses, 1 out of 6, 2 out of 11 and 3 out of 10 showed a worsening in lameness grade at 3, 12 and 24 months respectively, after a previous lameness improvement. In horses with baseline lameness grade 2 (n = 14), 62% were non-lame at 1 month, and 79 to 100% were non-lame at the following controls (3, 6, 12 and 24 months). In horses with baseline lameness grade 3 (n = 15), 50% and 62% were non-lame at 1 and 3 months respectively, whereas 80 to 87% were non-lame at the following controls (6, 12 and 24 months). In horses with baseline lameness grade 4 (n = 3), at 1, 3 and 6 months, only 1 horse was non-lame and 2 other horses were non-lame at 12 and 24 months. No lameness worsening was observed in horses with baseline grade 2, 3 and 4.

**Figure 1:**
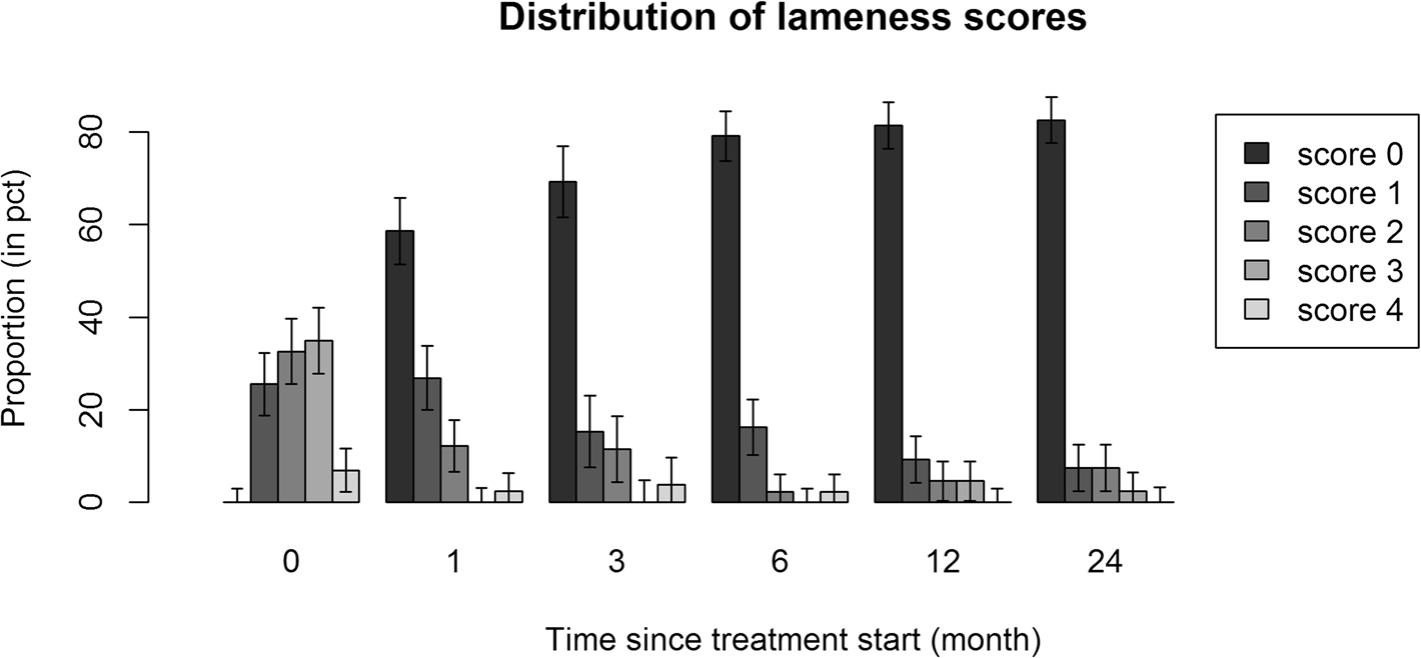
Distribution of lameness grades at baseline and at 1, 3, 6, 12 and 24 months following the treatment with PAAG. Error bars show standard errors of the estimated proportions within each time group. The left most bars in each time group correspond to non-lame horses. There was a significant increase in the proportion of non-lame horses between baseline and 1 month, followed by a steady increase between 3 and 6 months, then a stabilization in the proportion of non-lame horses between 6 and 24 months.

**Figure 2:**
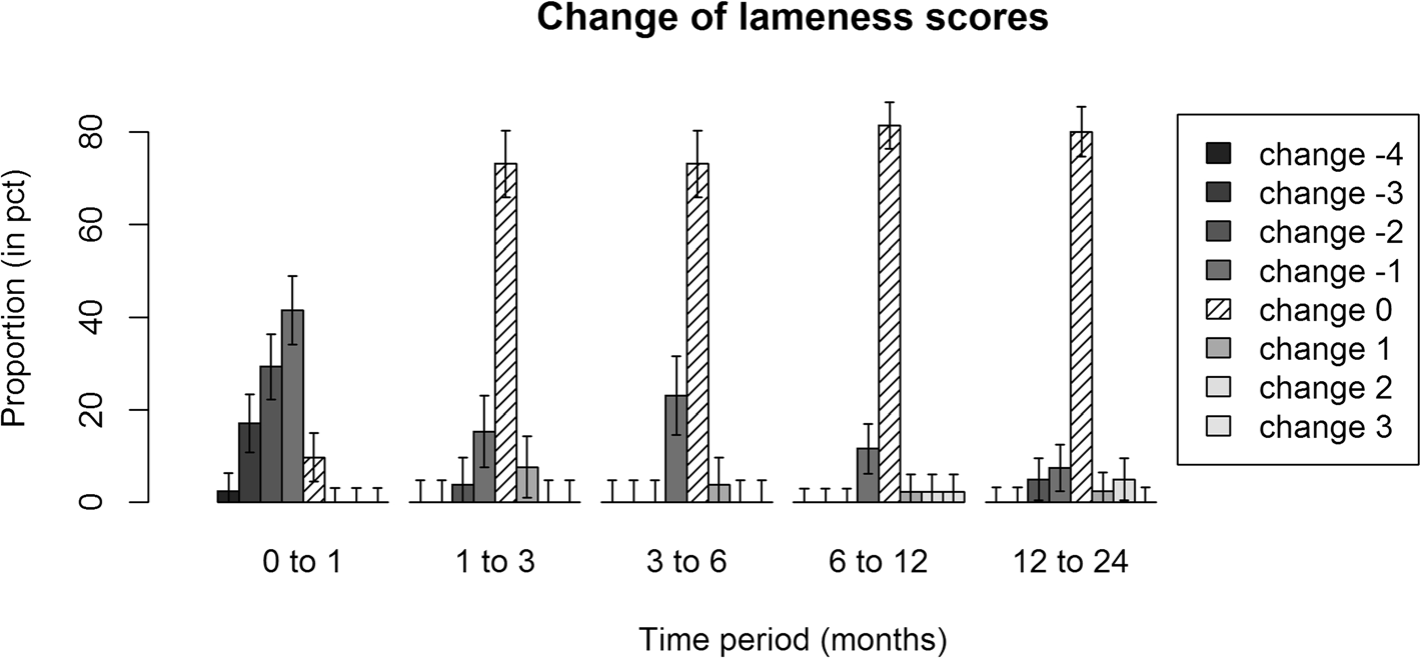
Distribution of change of lameness grades of individual horses over consecutive time points. Error bars show standard errors of the estimated proportions within each time period. The middle bars with shading lines correspond to horses that have retained their lameness score over the designated time period. The largest reduction in lameness took place at 1 month follow-up.

There was a significant increase in the proportion of non-lame horses between baseline and 1 month, followed by a steady increase between 3 and 6 months, then a stabilization in the proportion of non-lame horses between 6 and 24 months (Figure 1). Concerning the outcome, at 1, 3, 6, 12 and 24 months follow-up, irrespective of the baseline lameness grade, 59%, 69%, 79%, 81/% and 82.5% of horses were non-lame respectively. Figure 2 shows the distribution of the change in lameness grades for the individual horses over the observed time periods. The largest reduction in lameness took place between baseline and 1 month follow-up. After 1, 3, 6 and 12 months follow-up, 73%, 73%, 81% and 80% of the horses respectively, retained the lameness grade at the following lameness evaluation.

At baseline, joint effusion grade was 0 (7%), 1 (23%), 2 (42%), 3 (16%) and 4 (12%), whereas at 24 months joint effusion grade was 0 (77.5%), 1 (20%), 2 (0%), 3 (2.5%) and 4 (0%). No adverse effects associated with the treated joints were detected during the study period.

At 24 months, 90% of the owners were satisfied or highly satisfied with the outcome of the treatment, whereas 10% of the owners were slightly satisfied or not satisfied with the outcome of the treatment.

The statistical analysis showed a highly significant reduction of the lameness grade (all grades) after baseline (*P* < 0.0001), and a highly significant association between lameness grade and joint effusion (*P* < 0.0001). Estimates for the odds ratios (OR) showed that OR for lower lameness from month 1 to month 24 relative to baseline increased from 20 (95% CI = 6–67) to 58 (95% CI = 12–275) (Table 3). These confidence intervals for OR were wide; however, they were clearly bounded away from 1, which was also reflected by the p-value. Thus, there was a highly significant effect. OR for lower lameness grade was 3.1 (95% CI = 2.0-4.9) when joint effusion decreased by one grade (Table 3).

**Table 3:** **Estimated odds ratios for lower lameness grade between time points and differences in joint effusion and associated 95% confidence intervals**

**Odds ratio**	**Estimate**	**95% confidence limits**	
**Month 1 vs Baseline**	20.45	6.24	66.98
**Month 3 vs Baseline**	23.16	5.65	94.85
**Month 6 vs Baseline**	50.94	12.29	211.05
**Month 12 vs Baseline**	66.64	15.46	287.14
**Month 24 vs Baseline**	57.71	12.12	274.71
**Effusion −1**	3.12	1.97	4.94

Odds ratios for lower lameness from month 1 to month 24 relative to baseline increased from 20 (95% CI = 6–67) to 58 (95% CI = 12–275).

Estimates for OR showed that joint effusion score decreased significantly over time (*P* < 0.0001), and decreased significantly with radiography scoring (*P* = 0.0041) (Table 4): OR for lower joint effusion grade from month 1 to month 24 relative to baseline increased from 13 (95% CI = 5–34) to 172 (95% CI = 46–637). These confidence intervals for OR were wide; however, they were clearly bounded away from 1, which was also reflected by the p-value. OR for lower joint effusion scoring was 3.1 (95% CI = 1.9-5.1) when radiographic grade at baseline was low by one grade.

**Table 4:** **Estimated odds ratios for lower joint effusion grade between time points and differences in radiography grade at baseline and associated 95% confidence intervals**

**Odds ratio**	**Estimate**	**95% confidence limits**	
**Month 1 vs Baseline**	13.21	5.14	33.96
**Month 3 vs Baseline**	38.18	11.72	124.40
**Month 6 vs Baseline**	106.14	32.31	348.61
**Month 12 vs Baseline**	91.52	28.19	297.07
**Month 24 vs Baseline**	171.80	46.35	636.76
**Radiography grade −1**	3.12	1.90	5.13

OR for lower joint effusion grade from month 1 to month 24 relative to baseline increased from 13 (95% CI = 5–34) to 172 (95% CI = 46–637). OR for lower joint effusion grade was 3.1 (95% CI = 1.9-5.1) when radiographic grade at baseline was low by one grade.

## Discussion

This 2 year clinical study demonstrated that PAAG significantly alleviated lameness in osteoarthritic joints, as assessed by clinical lameness evaluation. A similar outcome was found in a recent pilot randomized controlled study on an experimental OA model in goats, where 75% (3 out of 4) of goats treated with PAAG were non-lame 4 months after the treatment [18]. No adverse effects were observed during the study period in the treated joints, which is consistent with previous studies using PAAG intra-articularly to treat equine OA [16,17,19,20]. PAAG has also proven to be safe in humans for more than 15 years of use [12-14].

The statistical analysis showed a highly significant (*P* < 0.0001) reduction of the lameness grade after baseline. The estimated OR showed an increased reduction over time from OR = 20 from baseline to month 1 to OR = 58 from baseline to month 24. The largest reduction in lameness grade appeared from baseline to month 1. After month 1 the lameness grade continued to decrease, although the difference between months 1, 3, 6, 12 and 24 was non-significant (p = 0.18). In particular, the OR was very constant from months 6 to 24. Thus, the clinical improvement in lameness grade was already present one month after PAAG treatment. This suggests that the effect of PAAG on OA might occur mainly during the first month after treatment and lasts and increases progressively until 6 months, with a stabilization between 6 and 24 months.

Worsening of the lameness grade following a previous lameness improvement was observed in only 3 horses with baseline lameness grade 1 (n = 3/11). Since no radiographic follow-up was performed in our clinical trial, it is difficult to speculate on the reason of lameness worsening in these horses. None of the horses with baseline lameness grade 2, 3 or 4 showed deterioration in lameness.

This clinical study has also demonstrated that joint effusion grade decreased significantly over time (*P* < 0.0001). At baseline, joint effusion was absent in only 7% of the horses, while at 24 months the majority of horses (77.5%) showed no joint effusion of the treated joints. Since lameness grade decreased significantly (*P* < 0.0001) with lower effusion grade, part of the lameness improvement over time can be seen through lowering of the joint effusion.

Although joint effusion was subjectively assessed in this study, PAAG induced a significant decrease in joint effusion in the osteoarthritic joints. However, the mechanism-of-action of PAAG in reducing joint effusion in osteoarthritic joints needs to be investigated.

The majority of horses (86%) had received a previous unsuccessful anti-osteoarthritic treatment, before receiving PAAG, but there was no correlation between the previous treatment and the outcome lameness variable. In some cases (14%), which were mainly among the last included cases, PAAG was used as a first line treatment based on the encouraging results of the first cases of the study.

At 24 months, 90% of the owners were either satisfied or very satisfied with the outcome of this new OA treatment. This is consistent with the outcome as assessed by the veterinary clinicians (82.5% of non-lame horses at 24 months).

Although conventional concepts of OA emphasize the direct and predominant involvement of cartilage and bone in OA development, it is increasingly recognized that the synovium also contributes to the central pathophysiological event of cartilage matrix depletion. Lack of joint lubrication is postulated to play a significant role in the pathogenesis of OA [24]. This emphasizes the role of viscosupplementation, and hence the improvement of lubrication within the joint, in protecting a joint suffering from OA, and reducing the resulting pain. Recently, a study supported the use of intra-articular lubricin as an adjunct to viscosupplementation for retarding cartilage degeneration and possibly the development of post-traumatic OA [25,26].

Precise characterization of the mechanism-of-action of PAAG on osteoarthritic joints has not yet been established, but histopathological observations on joint tissue from horses [Christensen L, personal communication] and goats [18] have demonstrated that PAAG, like in other soft tissues, becomes integrated within the synovial membrane.

In the goat study [18], the synovial membrane of the joints injected with PAAG had a better elastance when compared to the synovial membrane of the control joints. Osteoarthritic joints typically show joint stiffness which is a major source of pain in OA. This is supported by a recent study on human knee joint stiffness, which showed that the stiffness co-efficient was higher in individuals with painful OA compared to those with normal knees [27].

By integrating the synovial membrane, which may probably decrease the joint capsule stiffness and hence the joint stiffness, PAAG might relieve pain of the osteoarthritic joint. This theory is supported by clinical observations in the study population where osteoarthritic joints that responded well to PAAG were no more painful to passive manipulation of the joints.

The inclusion criteria in the present study were strict in order to maximize the validity of the results. Nevertheless, there were some study limitations including a low number of horses, the fact that it was a prospective non controlled clinical study, and the subjective assessment of joint distension. A quantitative measurement of joint circumference could have been performed. This was a multi-centre study, which represented another study limitation due to several clinicians involved in the study, and the potential for inconsistency in application of the lameness grading scale among the clinicians and within clinicians at different examinations [28]. In addition, radiography was not used for the follow-up of OA because of its association with a series of concerns including the insensitivity of radiographs to detect early and small changes and the slow progression of OA being a common finding in clinical trials [5]. Repeatability of application of the radiographic grading system was not assessed.

The present study has shown that PAAG relieved or completely removed the symptoms of lameness and the joint distention in osteoarthritic joints and can be considered as a disease-modifying OA therapeutic agent. A recent study on an OA model in goats [18] has shown that PAAG reduces the progression of OA as evaluated by MRI and histopathology, which supports the hypothesis that PAAG contributes to a disease-stabilizing affect. Further work investigating the mechanism of action of PAAG in osteoarthritic joints is required.

## Conclusions

PAAG significantly alleviated lameness and joint effusion in osteoarthritic joints in horses. PAAG is a promising, safe and lasting (at least 24 months) new treatment for OA in horses and its further evaluation is warranted.

### Endnote

^a^ Arthramid® Vet, Contura International A/S, DK-2860 Søborg, Denmark.
